# Burden of multidrug-resistant organisms in Hungary

**DOI:** 10.1186/1753-6561-5-S6-P297

**Published:** 2011-06-29

**Authors:** E Szilágyi, Á Tóth, Z Végh

**Affiliations:** 1 Epidemiology, Office of the Chief Medical Officer, Budapest, Hungary; 2 Bacteriology, National Center for Epidemiology, Budapest, Hungary

## Introduction / objectives

Infections caused by multidrug-resistant organisms (MDROs) increase morbidity, mortality, lengths of hospital stay and health care costs, therefore surveillance and infection prevention and control measures are crucial.

## Methods

The National Bacteriological Surveillance System (NBS) in Hungary was established with the aims to monitor resistance rates and trends of the most important pathogens in invasive isolates, in all inpatient and in outpatient isolates. The database of NBS includes more than 65% of all positive results of clinical bacteriological samples published by Hungarian laboratories. We monitored resistance rate trends for most frequent nosocomial pathogens between 2003 and 2010.

## Results

The most prevalent MDROs in Hungary proved to be Meticillin-resistant *Staphylococcus aureus* (MRSA), 3rd generation cephalosporin resistant *Klebiella pneumoniae* (CR-KP) and 3rd generation cephalosporin resistant *Escherichia coli* (CR-EC). The rate of MRSA in invasive isolates increased from 15% in 2003 to 26% in 2010. CR-KP rate in invasive isolates was 10% in 2003 and increased to 49% in 2010, while CR-EC in invasive isolates increased from 1% in 2003 to 21,5% in 2010. A similar steep increase in resistance rates has been observed in all inpatient and outpatient isolates for all the three pathogens.

## Conclusion

During the last several years, the prevalence of MDROs in Hungarian hospitals has increased steadily. As hand hygiene is the most simple, efficient and cost-effective measure to prevent healthcare associated infections, we launched our National Hand Hygiene Campaign in March 2011. The aims of our campaign are to promote and improve hand hygiene in Hungarian hospitals and hereby to reduce hospital infections and MDROs in Hungary.

## Disclosure of interest

None declared.

